# Sleep Deprivation Adversely Impacts Resident Performance for Simulated Arthroscopy

**DOI:** 10.1016/j.asmr.2021.04.001

**Published:** 2021-07-09

**Authors:** Quentin Baumann, Yassine Bulaid, Axel Van Vliet, Antoine Gabrion, Céline Klein, Patrice Mertl

**Affiliations:** a Department of Orthopedic and Trauma Surgery, SOS Mains, Amiens University Medical Center and Jules Verne University of Picardie, Amiens cedex 1, France; b Department of Pediatric Orthopedic Surgery. Amiens University Medical Center and Jules Verne University of Picardie, Amiens cedex 1, France; c Orthopedic and Sports Surgery Center, Cap Ortho, Clinique Anne d’Artois, Bethune, France

## Abstract

**Purpose:**

The purpose of the study was to assess the performance of residents in orthopaedics before and after a 24-hour shift on a shoulder arthroscopy simulator. The primary study endpoint was an overall performance score (OPS) generated by the simulator.

**Methods:**

A prospective, comparative study of 120 simulator trials by 10 resident junior surgeons was performed in our university hospital’s simulation center between May and November 2018. To avoid memorization bias, all participants performed the same exercise 10 times on a VirtaMed ArthroS simulator prior to the study. Each resident’s performance (the OPS, the operating time, the proportion of procedures with iatrogenic lesions, the camera path length and the hook path length) in two different simulated arthroscopy exercise tasks was assessed once before and once after a 24-hour shift. This sequence was performed three times during the semester, and the change over time in performance was also evaluated.

**Results:**

The OPS was significantly lower after the night shift (*P* = 0.035 for the first exercise, and *P* = 0.025 for the second).

**Conclusion:**

In a group of previously trained resident junior surgeons, overall performance with an arthroscopy simulator was significantly worse after a 24-hour shift. The study of secondary parameters of the OPS and the subgroup analysis based on the sleep time and Epworth score vary depending on the type of exercise performed arthroscopically. However, the use of a simulator after a night shift did not prevent the trainee from improving his/her level of performance over time.

**Level of Evidence:**

II, a prospective, comparative study

## Introduction

The combination of great demand for care and the low availability of medical resources has always prompted physicians to work selflessly beyond their physical limits.^1^^,^^2^^,^^3^ Executing surgical procedures after a night shift is still common practice, especially in services with mixed activity: planned surgery and trauma. It contributes to the heroism of the health professions—but this can have consequences on the patient^4^—and to medical education.^5^

Medical education has been enriched by the development of simulators. New parameters can be recorded digitally, and data are collected easily. Howells et al.^6^ established that arthroscopy skills acquired on a simulator were indeed transferred to procedures in the operating room—confirming that levels of performance with experimental models can be extrapolated to real conditions. Thus, simulators are acknowledged to be effective tools for teaching anatomy without resorting to cadaver specimens, which are scarce, expensive, and subject to burdensome regulations.^7^, ^8^, ^9^ It is now possible to carry out ethically sound, low-cost studies of the quality of teaching in surgery without jeopardizing patient safety.^10^

Hence, the study’s primary objective was to assess whether the overall performance score (OPS) on an arthroscopy simulator after vs before a 24-hour shift differed significantly. The secondary objectives were to determine *1*) which exercises and skills were modified by having worked a night shift, and *2*) whether performance with the arthroscopy simulator improved over time.

The purpose of the study was to evaluate the impact of a 24-hour working shift on the performance of orthopaedic residents during simulated arthroscopic exercises

We hypothesized that a night shift would reduce the level of performance achieved on an arthroscopy simulator.

## Methods

### Participants

The study prospectively included 10 residents in orthopedic surgery. Inclusion criterion was residents of the department who gave their consent to participate in the study. There were no exclusion criteria.

To avoid memorization bias, each participant practiced the study exercises 10 times in our university hospital’s simulation center before being included in the study. The participants were instructed not to drink caffeine-containing drinks or take any psychoactive substances during the 24-hour shift. The number of hours slept by the participants was noted, and the participants filled out the Epworth Sleepiness Scale (ESS) self-questionnaire^11^ (giving a score that ranged from 0: no tiredness, to 24: maximum tiredness) for the night before after the 24-hour shift and the night during the 24-hour shift. During 6 months in 2018 every 2 months they performed the protocol 1x session before and 1x session after the night shift (May–Nov) with the same people every 2 months. In this free interval, they did not train themselves on the simulator.

All participants were volunteers and were free to withdraw from the study at any time. According to French legislation, approval by an institutional review board was not required for studies that do not include patients.

### The 24-Hour Shift

The residents’ 24-hour shift was performed in the Trauma Department at our University Medical Center. Work during the shift included the admission of trauma patients referred from the emergency department or surgical units, the management of hospitalized patients, participation in trauma surgery as a junior surgeon, and organization of the morning staff meeting (presentation of newly hospitalized patients or patients having undergone surgery during the night). When possible, residents were able to sleep in an on-call room.

### Simulation

A right shoulder simulator (ArthroS, VirtaMed, Schlieren, Switzerland) was used to perform the protocol. Each session included the completion of two exercises the day before the 24-hour shift and then within an hour of the end of the shift. The 10 residents carried out three assessment sessions, with a one-month interval between each session.

The first exercise was called “catch the stars” (CTS), which consisted of finding five virtual stars inside the glenohumeral space within a given time. The operator then had to remove the stars from the joint without damaging the surface of the humeral or glenoidal cartilage. The second exercise was simulated subacromial decompression (SD), which more closely resembled a real operation. Each participant was asked to inspect a right shoulder, identify 20 anatomic landmarks and then to perform lateral acromioplasty with a virtual acromionizer. At the end of the simulations, the participant was given a composite OPS, with between 0 and 60 points for the CTS and between 0 and 140 for the SD. The OPS was used as the primary outcome measure before and after the shift. The OPS included points for the operating time, the visualization of each anatomical structure as a percentage of the total, the camera path length, the hook or acromionizer path length, and the proportion of the surface area of the glenoid and the humeral head damaged during the exercise. Each of these component variables was studied as a secondary endpoint. During the simulations, the participant did not receive help from third parties (i.e., other physicians or from the simulator’s exercise manager). 10 residents participated in 3 simulator sessions every 2 months for 6 months. One session consisted of performing two exercises CTS and SD at 8:00 a.m. and the same exercise at 9:00 a.m. the day after) for 2 exercises per 3 sessions per 10 residents. For a total of 120 exercises analyzed.

Y.B., a senior surgeon of the department, was present during the evaluation. For each session, the average learning curve was collected, so that different sessions could be tracked.

### Statistical Analysis

All statistical analyses were performed with Excel for Mac 16.16.7 software (Microsoft, Redmond, WA) and RStudio software (RStudio PBC, Boston, MA). According to the systematic review of literature of Hetaimisch,^1^ the number of participants in these studies were between 9 and 42 participants. The repetition of three sessions made it possible to increase the number of evaluations on a self-paired population.

A Shapiro-Wilk test was used to determine whether data were normally distributed. The results were quoted as the mean [95% confident interval (CI)]. A paired Student’s *t*-test was used to assess before vs after differences for a given participant. A nonparametric Mann-Whitney *U*-test was used to compare values of quantitative variables. Spearman’s coefficient was calculated in order to assess correlations between qualitative variables and quantitative variables. The threshold for statistical significance was set to *P* < .05.

In a subgroup analysis, participants were divided into two equal groups according to the median sleeping time during the shift (group A: > 3 h (*n* = 5 for each of the three sessions, i.e., 15 in total); group B: <3 h (*n* = 15)) or the median ESS (group C: ESS ≤ 7 (*n* = 15); group D ESS>7 (*n* = 15)). A Mann-Whitney *U* test was used to differences between these subgroups. A paired Student’s *t*-test was used to compare the mean OPS and mean values of secondary parameters after vs before the shift.

## Results

There were 7 males and 3 females with a mean (range) age of 28.2 years (25-30 years) included in the study. The mean (range) number of semesters spent in an orthopedic surgery department was 6.8 (2–10). On average, residents had performed 1.2 (range: 0–10) arthroscopies as the main operator in the previous 12 months. Only two of the 10 study participants had a university diploma in arthroscopy.

### Overall Performance Before and After a Night Shift

On the night before the 24-hour shift, the mean (range) sleeping time was 5.8 hours (2.5–7) and the mean (range) ESS was 5.53 (3–10). The mean sleeping time during the shift was 3.3 hours (0–7): with an ESS of 12.5 (4–21). The mean OPSs for each exercise are detailed in Table 1. The performance was significantly better before the shift than after the shift (*P* < .04 and .02 for the CTS and the SD exercises, respectively).

**Table 1:** The mean ± SD (range) OPS before and after a 24-hour shift (P < .05 in a paired Student’s t-test)

	Before the Shift	After the Shift	*P*
“Catch the stars” exercise	48.83 ± 5.22 (18–60)	43.23 ± 5.69 (10–60)	.038
Subacromial debridement exercise	124.34 ± 3.84 (97.2–136)	118.64 ± 5.52 (76.2–135)	.025

OPS, Overall Performance Score.

### Secondary Parameters Before and After a Night Shift

The secondary outcomes composing the OPS are summarized in Table 2. In the CTS exercise, the proportion of glenoid cartilage surface area damaged during the exercise was significantly greater after the 24-hour shift (*P* = .03). The camera path length, the hook path length and operating time were also significantly greater after the 24-hour shift (*P* < .01 for all). In the SD exercise, the proportion of the glenoid and humeral cartilage surface areas damaged during the exercise before and after the shift did not differ significantly (*P* = .87 and *P* = .13). The same was true for the camera path lengths (*P* = .13), the acromionizer path length (*P* < .44) and the operating time (*P* < .77).

**Table 2:** Secondary parameters constituting the OPS before and after the shift, compared with using a paired Student’s t-test

	Before the Shift	After the Shift	*P*
	Catch the Stars		
Glenoid lesion	.77 ± .27 (0–3)	1.7 ± .64 ((0–7)	.03
Humerus lesion	1.5 ± .56 (0–5)	2.37 ± .92 (0–13)	.11
Camera path length (cm)	56.61 ± 11.44 (2.3–132.1)	84.11 ± 21.12 (20.8–230)	.01
Grasper path length (cm)	153.11 ± 27.15 (70.5–357.6)	208.50 ± 43.42 (91.2–533.3)	.02
Time (s)	99.3 ± 15.29 (38–192)	153.07 ± 29.43 (55–383)	<.01
	Subacromial Decompression		
Glenoid lesion	2.73 ± .62 (0–7)	2.8 ± .8 (0–11)	.87
Humerus lesion	4.53 ± .91 (1–10)	5.2 ± .98 (0–12)	.13
Camera path length (cm)	266.50 ± 44.37 (113.4–557.2)	362.38 ± 120.82 (142.9–1887.1)	.13
Acromionizer path length (cm)	237.96 ± 61.67 (41.5–61.5)	221.22 ± 45.09 (76.4–565)	.44
Time (s)	373.23 ± 75.04 (181–937)	364.27 ± 45.6 (181–602)	.77

CTS, “catch the stars”; SD, subacromial decompression.

### Subgroup Analysis as a Function of Sleep and Sleepiness During the 24-Hour Shift

#### Sleeping Time

A subgroup analysis of performance in the CTS and SD exercises with regard to the median sleeping time (3 h) during the night shift did not show any significant differences between groups A and B in the OPS, glenoid lesions, humeral lesions, camera path length, acromionizer path length, grasper path length, or completion time (Table 3).

**Table 3:** Subgroup analysis as a function of the median sleeping time during the 24-hour shift

	Group a Sleeping Time < 3 h	Group B Sleeping Time > 3 h	*P*
	*n* = 15	*n* = 15	
	CTS Exercise		
OPS	40.33 ± 16.08 (34.33–46.33)	46.13 ± 14.27 (38.23–54.04)	.10
Glenoid lesion (%)	1.4 ± .69 (0–3)	2 ± 1.08 (0–7)	.59
Humerus lesion (%)	1.93 ± .87 (0–5)	2.8 ± 1.65 (0–13)	.52
Time (s)	149.87 ± 36.82 (75–290)	155.6 ± 48.2 (55–383)	.90
Camera path length (cm)	81.54 ± 27.32 (29.5–187.7)	86.67 ± 35.81 (20.8–230)	.87
Grasper path length (cm)	200.11 ± 55.94 (101.2–471.1)	216.9 ± 73.58 (91.2–533.3)	.98
	SD Exercise		
OPS	121.31 ± 5.67 (99–134)	123.93 ± 3.27 (114–135)	.90
Glenoid lesion (%)	3.07 ± 1.36 (0–11)	2.53 ± .9 (0–5)	.73
Humerus lesion (%)	5.53 ± 1.68 (0–12)	4.87 ± 1.08 (1–8)	.54
Time (s)	390.53 ± 66.43 (241–602)	338 ± 62.75 (181–602)	.25
Camera path length (cm)	434.39 ± 244.05 (152.2 – 1887.1)	290.37 ± 59.49 (142.9–543.2)	.82
Grasper path length (cm)	257.6 ± 79.70 (83.6–565)	184.83 ± 45.18 (76.4–401-.2)	.23

A paired Student’s *t*-test was used for all comparisons except that of the acromionizer path length, in which a Mann-Whitney *U*-test was applied. CTS, “catch the stars”; OPS, overall performance score; SD, subacromial decompression.

#### Epworth Sleepiness Scale

The results of the subgroup analysis with regard to the median ESS (group C ≤7 vs group D >7) are summarized in Table 4. For the CTS exercise, there were no significant intergroup differences with regard to the OPS, glenoid lesions, humeral lesions, camera path length, acromionizer path length, grasper path length, and completion time. For the SD exercise, the mean ± SD (range) OPS was significantly higher in group C [125.93 ± 3.36 (99-136)] than in group D [117.05 ± 5.53 (76.20-134); *P* =.003].

**Table 4:** Subgroup analysis as a function of the median ESS during the 24-hour shift

	Group C ESS ≤ 7	Group D ESS > 7	*P*
	*n* = 15	*n* = 15	
	CTS Exercise		
OPS	47.43 ± 5.98 (10–60)	44.63 ± 5.05 (12–60)	.094
Time (s)	104.57 ± 24.34 (38–383)	147.8 ± 24.1 (41–290)	.003
Glenoid lesions (%)	0.83 ± .5 (0–7)	1.63 ± 0.5 (0–5)	.005
Humeral lesions (%)	1.63 ± .95 (0–13)	2.23 ± 0.54 (0–5)	.018
Camera path length (cm)	65.3 ±18.31 (20.8–230)	75.42 ± 17 (29.5–187.70)	.15
Grasper path length (cm)	162.13 ± 37.37 (70.5–533.5)	199.48 ± 36.7 (87–471.1)	.035
	SD Exercise		
OPS	125.93 ± 3.36 (99–136)	117.05 ± 5.53 (76.20–134)	.003
Time (s)	369.53 ± 75.21 (190–937)	367.97 ± 45.38 (181–602)	.35
Glenoid lesions (%)	2.37 ± .66 (0–7)	3.17 ± .73 (0–11)	.073
Humeral lesions (%)	4.57 ± .93 (1–10)	5.17 ± .97 (0–12)	.382
Camera path length (cm)	254.28 ± 40.68 (113.4–557.2)	374.61 ± 12054 (142.1–1887.1)	.028
Acromionizer path length (cm)	233.02 ± 59.96 (41.5–615)	226.15 ± 47.52 (76.4–565)	.784

A paired Student’s *t*-test was used for all comparisons except that of the acromionizer path length, in which a Mann-Whitney *U*-test was applied. ESS, Epworth Sleepiness Score; CTS, “catch the stars”; OPS, overall performance score; SD, subacromial decompression.

### Assessment of the Learning Curve Following the Pre- or Post-Call Status

**Fig 1:**
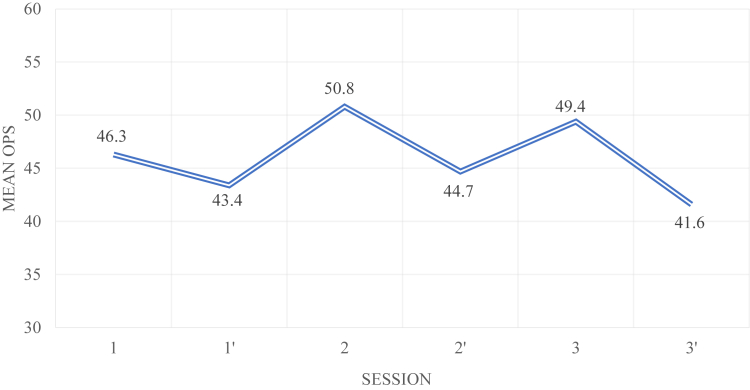
Change over time in the mean OPS for the CTS exercise, before (1, 2, 3) and after (1’, 2’, 3’) the shift (three sessions).

**Fig 2:**
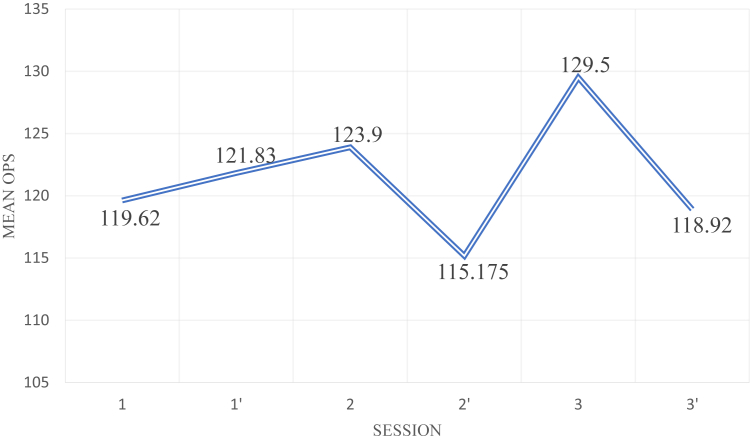
Change over time in the mean OPS for the SD exercise, before (1, 2, 3) and after (1’, 2’, 3’) the shift (three sessions).

The changes over time in before-and-after differences in the mean OPSs are shown in Figs 1 and 2. For the CTS exercise, performance was always worse after the shift. The same was true for the SD exercise, except for the first session. The data also show that for the CTS exercise, the mean “before-shift” OPS in the third session did not differ significantly from the mean “before-shift” OPS in the first session. For the SD exercise, the improvement was notable; the mean “before-shift” OPS in the third session was significantly higher than the mean “before-shift” OPS in the first session.

Our results are in line with most studies of larger numbers of orthopedic residents, which evidenced a negative impact of fatigue and sleep deprivation on performance in virtual reality simulators.^12^^,^^13^ The before vs after differences in the secondary endpoints composing the OPS (operating time, path lengths, iatrogenic lesions, etc.) for the two exercises were heterogeneous and did not enable us to detect overall trends in these parameters.

According to the secondary parameters constituting the OPS: in the CTS exercise (the most “fun” exercise, and the most removed from actual clinical situations), the completion time and the percentage of glenoid damage were both significantly higher after the 24-hour shift. In the SD exercise (which most resembles actual surgery), there were no before vs after differences.

## Discussion

The present results in this study confirmed our hypothesis: in two different exercises, we observed significantly lower performance after a 24-hour shift. Similar data can be found in the literature on orthopedic surgery and other surgical specialties, although the results depend on the techniques and methods used. From a methodological viewpoint, Yi et al.’s study of a laparoscopy simulator (LAP Mentor, Simbionix, Beit Golan, Israel) most closely resembles our present work. The researchers did not evidence a difference in the participants’ skills after a work shift.^14^ However, Yi et al. studied only 9 trainees and a single before vs. after session.^14^ Leu et al. studied the impact of sleep deprivation on simulated laparoscopic surgery performance among 20 novices (i.e., medical students and non-healthcare professionals without any experience of surgery).^15^ After 20 hours of sleep deprivation, no differences were found.^15^

One explanation for these results would be that the more realistic exercise prompted the residents to concentrate more when they were tired, as suggested by Al-Ecq et al.^16^

Our subgroup analysis as a function of the median sleep time during the shift did not reveal any significant difference in the OPSs. However, an ESS score >7 was associated with a significantly lower OPS after the shift in the SD exercise. This subgroup analyses lacked statistical power and would be interesting to repeat in a larger cohort. However, this finding might suggest that in on-call residents, the ESS is a better marker of fatigue than sleep time.

Very few studies have quantitatively and objectively assessed the learning curve for shoulder arthroscopy.^17^^,^^18^ This operation is reputed to be difficult, with a very steep learning curve; however, the plateau phase has not been well defined. The difficulty of a surgical exercise appears to be correlated with the time it takes for a trainee to reach the plateau. For example, Manuel-Palazuelos et al.’s study found that the plateau phase for gastro-jejunal anastomoses using a laparoscopy simulator was about 20 procedures.^19^ In the present study, we sought to prevent memorization bias by asking residents to perform each of the two exercises 10 times (a number chosen arbitrarily) before their inclusion in the test protocol. Thus, in the (easier) CTS exercise, we did not observe an improvement in the preshift OPS between the first session and the third session—suggesting that the plateau phase had been reached. Walbron et al. also evaluated residents in the CTS exercise, using the same simulator as in the present study.^20^ The researchers did not report on a learning curve for the OPS, although the performance in terms of time, camera path length, and grasper path length were still increasing after six trials.

Subacromial decompression is a more technically challenging exercise. We observed an improvement in the pre-shift OPS between the first session and the third session, which suggests that the learning plateau had not been reached.

Furthermore, participating in a simulator training session after a 24-hour shift call was not associated with poor performance in the following session. The benefits of repeating simulation have been extensively described in the literature.^20^, ^21^, ^22^ Our results relate to the use of simulators after a long shift, since this approach does not appear to prolong the learning curve.

Initially, a reduction in the residents’ weekly working time and the need for supervision of the residents’ work after a call was met with suspicion by the medical center’s program directors. They feared that a reduction in residents’ working time would have a negative impact on the acquisition of professional skills, experience in the operating theater, and the continuity of care provision in their department.^23^

However, the benefits of a reduction in working time are already apparent, such as the number of scientific publications published by residents during their residency program,^24^ and an improvement in residents’ quality of life. The results of the present study suggest that time spent outside of the hospital can be used for simulation training.

### Limitations

Our study had several limitations. First, it had a single-center design. Second, we did not study the influence of the number of years of residency—in contrast to the work by Martin et al., Howell et al., and Rebodo et al.^4^^,^^21^^,^^25^

The numbers of participants (*n* = 10) and sessions (*n* = 3 in total) included in the present study were small but are not dissimilar to those found in the literature on similar topics. In Aïm et al.’s systematic review, it was reported that simulator studies involved an average of 30 trials (range: 7-78).^26^ One of the strengths of our study was its analysis of three different sessions. Moreover, the study’s single-center design meant that all the participants had received the same surgical training.

Furthermore, the pairing was well matched because each resident acted as his/her own control in before vs. after comparisons.

Our results for the secondary endpoints also revealed important data: the residents’ mean nightly sleeping time even before a 24-hour shift (mean: 5.8 hours) was well below the American Academy of Sleep Medicine and the Sleep Research Society’s recommendation (7 to 9 hours). ^27^ Our observation is in line with Sochacki et al.’s report.^28^ This might have led to bias and underestimation, since our participants were not "fully" rested during the preshift evaluation. A further study strength was our evaluation of postshift performance during the 25th hour, i.e., immediately after the end of the shift. It has been shown that performance in a virtual reality simulator improves when the exercise is repeated within 48 hours of the initial session.^20^ However, we observed a significantly lower OPS after the 24-hour shift; this suggests that working a night shift has a negative effect on arthroscopy skills. Another source of bias might have been differences in the nature of the night shift from one study to another or within a study; one can reasonably assume that shift involving operations in the middle of deep night and/or challenging surgical procedures induces more fatigue than an equivalent shift in which the surgeon gives emergency advice and sets casts. Although we recorded the ESS as an index of fatigue, other factors may have influenced our results.

Lastly, our assessment of the learning curve might have prompted firmer conclusions if we had included a control group of nonfatigued participants who were not tested after a 24-hour shift.

### Conclusions

In a group of previously trained resident surgeons, overall performance with an arthroscopy simulator was significantly worse after a 24-hour shift. The study of secondary parameters of the OPS and the subgroup analysis based on the sleep time and Epworth score vary depending on the type of exercise performed arthroscopically. However, the use of a simulator after a night shift did not prevent the trainee from improving his/her level of performance over time.

## Supplementary Data

ICMJE author disclosure forms
